# Genetic Dissection of End-Use Quality Traits in Adapted Soft White Winter Wheat

**DOI:** 10.3389/fpls.2018.00271

**Published:** 2018-03-09

**Authors:** Kendra L. Jernigan, Jayfred V. Godoy, Meng Huang, Yao Zhou, Craig F. Morris, Kimberly A. Garland-Campbell, Zhiwu Zhang, Arron H. Carter

**Affiliations:** ^1^Department of Crop and Soil Sciences, Washington State University, Pullman, WA, United States; ^2^Western Wheat Quality Laboratory, Agricultural Research Service, United States Department of Agriculture, Pullman, WA, United States; ^3^Wheat Health, Genetics, and Quality Research Unit, Agricultural Research Service, United States Department of Agriculture, Pullman, WA, United States

**Keywords:** association mapping, soft white wheat, end-use quality, Pacific Northwest, linkage disequilibrium, plant breeding

## Abstract

Soft white wheat is used in domestic and foreign markets for various end products requiring specific quality profiles. Phenotyping for end-use quality traits can be costly, time-consuming and destructive in nature, so it is advantageous to use molecular markers to select experimental lines with superior traits. An association mapping panel of 469 soft white winter wheat cultivars and advanced generation breeding lines was developed from regional breeding programs in the U.S. Pacific Northwest. This panel was genotyped on a wheat-specific 90 K iSelect single nucleotide polymorphism (SNP) chip. A total of 15,229 high quality SNPs were selected and combined with best linear unbiased predictions (BLUPs) from historical phenotypic data of the genotypes in the panel. Genome-wide association mapping was conducted using the Fixed and random model Circulating Probability Unification (FarmCPU). A total of 105 significant marker-trait associations were detected across 19 chromosomes. Potentially new loci for total flour yield, lactic acid solvent retention capacity, flour sodium dodecyl sulfate sedimentation and flour swelling volume were also detected. Better understanding of the genetic factors impacting end-use quality enable breeders to more effectively discard poor quality germplasm and increase frequencies of favorable end-use quality alleles in their breeding populations.

## Introduction

Kernel texture (hardness), water absorption, protein (gluten) strength, and milling quality differentiate the end-use quality of wheat (*Triticum aestivum* L.). Generally, hard wheat flours have higher gluten strength, damaged starch, and non-starch polysaccharides that lead to increased water absorption capacity, whereas soft wheat flours have lower gluten strength, damaged starch, and non-starch polysaccharides that lead to decreased water absorption capacity (Hoseney, 1994; Souza et al., 2002). Since phenotypic laboratory milling and baking quality assays are destructive, laborious, and expensive, breeders routinely do not use these methods for selection in early generations. Higher throughput and more cost-effective methods of screening early generation material are needed, such as smaller-scale phenotyping tests or use of molecular markers.

End-use quality traits in soft wheat are predominately controlled by genetic factors, so potential gains from selection in earlier generations is possible (Smith et al., 2011; Carter et al., 2012; Souza et al., 2012). Even though several major loci affecting wheat end-use quality have been characterized, numerous smaller effect quantitative trait loci (QTL) influence these traits and remain uncharacterized. Kernel texture, along with protein strength, are key criteria for wheat market classes and end-use. Hardness is primarily controlled by allelic variation in the *Pina* and *Pinb* genes in the *Ha* locus on 5DS (Bhave and Morris, 2008a,b). However, soft wheats are fixed for the wild type alleles at both *Pina* and *Pinb*, so smaller effect genetic factors from other genomic regions impact variation in hardness in this market class (Morris, 2002; Kiszonas et al., 2013a). Flour protein content, damaged starch, and non-starch polysaccharides affect water absorption and dough rheology.

Gluten is comprised of both glutenins and gliadins which function as endosperm storage proteins. Glutenins are controlled by the high molecular weight (HMW) and the low molecular weight (LMW) loci on the long and short arms of chromosome 1A, 1B and 1D (Payne et al., 1987; Ibba et al., 2017). The HMW glutenins (encoded by *Glu-1*), and their corresponding x- and y-type subunits account for 47-60% of the variation in bread making quality of wheat (Payne et al., 1987; Lukow et al., 1989). The genes for gliadins are found on homoeologous groups 1 and 6 chromosomes (Payne et al., 1984; Payne, 1987). The genes that encode the granule-bound starch synthase1 (GBSS1) enzyme (*Wx-4A, Wx-7A*, and *Wx-7D*) on chromosomes 4A, 7A, and 7D control starch amylose composition in wheat, a key factor in white salted noodle texture (Nakamura et al., 1993; Epstein et al., 2002). Non-starch polysaccharides exert a large influence upon water absorption and dough rheology despite being minor flour constituents (Kiszonas et al., 2013b). Non-starch polysaccharides, mostly arabinoxylans, can oxidatively cross-link to increase dough water holding capacity (Izydorczyk and Biliaderis, 1992). While these major genes exert influence upon wheat end-use quality, they tend to be fixed in breeding population, especially by market class. Identification of the molecular markers associated with genetic factors contributing to soft wheat end-use quality will enable breeders to select for end-use quality in early generation advancement when grain is limited, but the number of lines is great. To date, the number of diagnostic molecular markers for quality traits is limited to the major genes for HMW, LMW, GBSS1 and puroindolines (Gale, 2005). DNA markers for the HMW *Glu-D1* locus that can discriminate between Dx2 + Dy12 and Dx5 + Dy10 genotypes are routinely in marker-assisted selection; the former being preferred for soft wheats and the latter for hard wheats due to its effect on gluten strength resulting in stronger dough and good bread making quality (Payne et al., 1981; Liu et al., 2008). Use of high-throughput marker assisted selection or genomic selection methods will facilitate evaluation of the high progeny numbers required to simultaneously improve end-use quality along with plant resistance to abiotic and biotic stresses, grain yield, and agronomic productivity.

Association mapping in germplasm collections is particularly useful for identifying common small effect QTL due to increased recombination events in the lineages of the accessions (Bernardo, 2008). The inbred lines commonly used for association mapping represent a wider genetic base of breeding materials than those used in genetic mapping of bi-parental populations; therefore, it should be possible to discern numerous genes of relative importance to the traits in question (Bernardo, 2008). When advanced generation breeding lines are used for association mapping, the favorable alleles associated with traits of interest may quickly become subjects of selection. Although there has been previous genome wide association mapping studies (GWAS) in soft wheat (Breseghello and Sorrells, 2006), there has not been a comprehensive report of kernel, milling and baking quality traits for soft white wheat. Since many of these traits are correlated (Souza et al., 2012), examining them in one population can provide evidence of pleiotropic loci. In addition, we examined one of only a few association mapping panels comprised of elite and adapted soft wheats.

It was hypothesized that significant marker-trait associations (MTAs) can be detected in a panel of soft white winter wheat adapted to the Pacific Northwest and that SNPs with pleiotropic effects on numerous end-use quality traits will also be identified. The objective of this study was to assess a regionally adapted germplasm panel for significant MTAs to several end-use quality traits. These results should further elucidate the genetic factors controlling soft wheat end-use quality.

## Materials and methods

### Germplasm

This association mapping panel was developed using 480 advanced soft white winter wheat breeding lines and released cultivars from Pacific Northwest breeding programs (Oregon State University, University of Idaho, Washington State University, USDA-ARS, and private breeding companies) selected from 1992 to 2014. Experimental lines were included, if they had at least six environmental observations in the Wheat Analysis System database from the USDA-ARS Western Wheat Quality Laboratory in Pullman, WA. The population contained lines with either a club or a lax head type. Club, Soft White (lax head), and Western White (a mixture of the two) are the three market sub-classes traded in commerce. Out of the 480 original lines in the panel, only 469 lines were eventually used in the association analysis. Three were phenotypically identified as hard white winter lines and an additional eight lines were excluded from the analysis due to poor DNA quality or missing marker covariate information. The degree of relatedness between these 469 lines is presented in Supplementary File 1.

### Phenotyping

Historic phenotypic data on all end-use quality traits were obtained from the Wheat Analysis System database in the USDA-ARS Western Wheat Quality Laboratory in Pullman, WA, USA. Environments were considered to include both crop year and field locations, for a total of 462 individual environments. These field locations included sites in Montana, Idaho, Oregon, and Washington from 1992 to 2014. Each genotype was represented by a single sample per environment. The dataset was unbalanced; not all experimental lines were grown in all environments, except for one location in 2014, where the entire association mapping panel was grown in one complete field replication in Pullman, WA.

Testing procedures from the American Association of Cereal Chemists International (AACC International, 2008) were used to measure the grain, milling, flour and baking quality traits (Table 1). Test weight was measured as grain weight per volume using Approved Method 55-10.01. The Single Kernel Characterization System (SKCS) 4100 (Perten Instruments, Springfield, IL, USA) was used to assess kernel size, weight, and hardness (Approved Method 55-31.01). Grain protein content was measured using near infrared reflectance (Approved Method 39-10.01). Flour protein content and flour ash content were measured using Approved Methods 39-11.01 and 08-01.01, respectively. All samples were milled on a Quadrumat Senior experimental mill (Brabender, South Hackensack, NJ, USA) as modified by Jeffers and Rubenthaler (1977). The milling traits measured included break flour yield, total flour yield and milling score. The milling score encompassed both total flour yield (FYELD) and flour ash (FASH) content in a single value (Carter et al., 2012). It was calculated as follows:

$$\begin{array}{l} MSCOR=(100-(0.5(16-13.0+\left(80-FYELD\right)\\ \;+50\left(FASH-0.30\right)))\times 1.274)-21.602 \end{array}$$

**Table 1:** Approved methods, units, means, and ranges of end-used quality traits in a Pacific Northwest soft white winter wheat diversity panel.

**Trait**	**Abbr**.	**Approved method**	**Units**	**Mean**	**Min**	**Max**	**S.E.^c^**	***h^2^*^d^**
**GRAIN CHARACTERISTICS**								
Kernel hardness	SKHRD	55-31.01	unitless	31.9	−4.1	55.7	0.13	0.89
Kernel size	SKSIZE	55-31.01	mm	2.6	1.7	3.3	<0.00	0.57
Kernel weight	SKWT	55-31.01	mg	36.3	22.1	57.9	0.07	0.61
Test weight	TWT	55-10.01	kg/hL	78.7	71	86.2	0.02	0.69
Grain protein	WPROT	39-10.01	percent	10.4	5.6	15.6	0.02	0.23
**MILLING TRAITS**								
Break flour yield	BKYLD	–	percent	49.2	32.4	55.8	0.04	0.80
Total flour yield	FYELD	–	percent	69.7	61.5	75.2	0.04	0.50
Milling score	MSCOR	–	unitless	85.6	68.3	98.5	0.06	0.52
**FLOUR PARAMETERS**								
Flour ash	FASH	08-01.01	percent	0.38	0.21	0.54	<0.00	0.47
Flour protein	FPROT	39-11.01	percent	8.7	4.2	13.7	0.02	0.29
Flour SDS^a^ sedimentation	FSDS	56-60.01	g/mL	9.5	1.6	27.3	0.07	0.68
Carbonate SRC	FSRC	56-11.02	percent	70.9	54.2	86.5	0.08	0.65
Lactic acid SRC	FSRL	56-11.02	percent	89.3	59.4	165.5	0.52	0.75
Sucrose SRC	FSRS	56-11.02	percent	90.2	69.5	124	0.17	0.46
Water SRC^b^	FSRW	56-11.02	percent	54.8	46.6	60	0.03	0.71
Flour swelling volume	FSV	56-21.01	mL/g	19.1	8.2	24	0.03	0.39
**BAKING PARAMETERS**								
Mixograph height	MPHT	54-40.02	cm	44.8	30.5	60.8	0.1	0.32
Mixograph width	MPW	54-40.02	cm	90.7	2	290.5	0.96	0.16
Mixograph width 2 mins	MPW2	54-40.02	cm	6.7	0.8	18.2	0.08	0.32
Mixograph peak time	MPTIME	54-40.02	min	2.3	0.5	7	0.02	0.16
Cookie diameter	CODI	10-52.02	cm	9.4	8.2	10.2	<0.00	0.71

a *SDS, sodium dodecyl sulfate*.

b *SRC, solvent retention capacity*.

c *S.E, standard error*.

d *h^2^, narrow-sense heritability*.

The sodium dodecyl sulfate (SDS) sedimentation test assessed gluten strength of flour samples (Approved Method 56-60.01), and the flour swelling volume test assessed the starch composition of flour samples (Approved Method 56-21.01). The four solvent retention capacity (SRC) tests assess different aspects of end-use quality using the following solvents: water, lactic acid, sucrose, and sodium carbonate. These different solvents provide insight into certain end-use quality traits: the water SRC measures overall flour absorption, the lactic acid SRC measures glutenin quality, the sucrose SRC measures non-starch polysaccharides and gliadins, and the sodium carbonate SRC measures damaged starch content (Gaines, 2000; Kweon et al., 2011).

Smaller-scale laboratory tests for dough rheology and overall end-use quality are especially useful to differentiate between the quality traits of later generation breeding lines. In soft wheat, sugar snap cookie diameter (Approved Methods 10-50.05) and Japanese sponge cake volume (Choi et al., 2012) are essential indicators of soft wheat end-use quality performance. Soft wheat flours with better overall quality will result in cookies with greater spread (diameter) and sponge cakes with greater volume (Slade and Levine, 1994; Choi et al., 2012; Kiszonas et al., 2015). The mixograph (Approved Method 54-40.02) measures the resistance of dough to overmixing, optimal gluten matrix development time, and other dough rheological properties. Mixograph measurements are useful because they provide insight into how an experimental line will perform in a commercial processing.

The different traits are grouped into four categories namely: grain characteristics, milling traits, flour and baking parameters (Table 1).

### Genotyping

The DNA for the association mapping panel was extracted as described by Naruoka et al. (2015). The association mapping panel was genotyped at the USDA-ARS Biosciences Research Laboratory in Fargo, ND, USA using an Illumina Infinium iSelect 90 K SNP chip, as specified by the manufacturer's protocols. A total of 81,575 markers were obtained from the 90 K SNP chip. GenomeStudio v2011.1 software (Illumina) was used for genotype classification and allele clustering. The default clustering algorithm was used to cluster each SNP into up to three allele clusters, then SNP clusters were manually curated to ensure more accurate genotyping. The SNP consensus map developed by Wang et al. (2014) was used to determine chromosome and chromosome position of SNPs. However, numerous unmapped SNPs were also included in the analysis.

### Statistical analyses

The historic phenotypic data were assessed for influential outliers (greater than 3 standard deviations) and these were removed prior to any statistical analyses. The summary statistics (minimum, mean, maximum and standard errors) were calculated for each phenotypic trait with Proc UNIVARIATE in SAS 9.3 (SAS Institute Inc., Cary, NC, USA) (Table 1). Due to the unbalanced phenotypic data included in the analysis, best linear unbiased predictions (BLUPs) for each phenotypic trait were calculated using Proc MIXED in SAS 9.3 with both genotype and environment as random effects. The mixed linear model was *y* = X + Zυ + *e*, where y is the vector of observations for an individual quality trait, X is the intercept, υ is the vector of random effects, Z is the design matrix for the random effects, and *e* is the vector of residuals. In our case, the genotype and environment effects were considered random and with only one observation per environment, the genotype by environment interaction served as an estimate of the residuals. Thus, genotype by environment interaction *per se* could not be evaluated in this dataset.

The BLUP for each trait were used as phenotypes in the final marker-trait association analysis. Narrow-sense heritability (*h*^2^) was calculated as the ratio of the additive variance to the total phenotypic variance using the R package rrBLUP (Endelman, 2011). Genome-wide annotated SNP markers were used determine the additive genetic variance of each trait and control for genetic relationships (Kruijer et al., 2015). Phenotypic correlations among traits were determined using Pearson coefficients calculated using JMP Genomics 6.0. Principal component analysis (PCA) was conducted to reduce the complexity of the correlation matrix for all end-use quality traits.

### Population structure and linkage disequilibrium (LD)

Linkage disequilibrium was calculated using 15,229 annotated markers and MAF greater than 5% in JMP Genomics 6.0. LD for markers on the same chromosome was measured using the squared allele-frequency correlation (*r*^2^) between alleles at two loci according to Weir (1996). A critical *r*^2^ value beyond which LD is due to physical linkage was determined by taking the 95th percentile of the *r*^2^ distribution for unlinked markers (Breseghello and Sorrells, 2006). Locally weighted polynomial regression (LOESS)-based curves were then fitted on scatter plots of r^2^ values plotted against the genetic distance (cM). The intersection of the LOESS curve fit and critical value of *r*^2^ was considered as the estimate of the extent of LD.

Since the association mapping panel was developed with different market sub-classes of wheat and from different breeding programs, population substructures were assumed to be present. Population structure was assessed using principle component analysis (PCA) calculated in GAPIT2 (Tang et al., 2016). Population differentiation was further assessed by estimating F_ST_ for individual loci (Wright, 1951) and genetic distance between subpopulations using the JMP Genomics 6.0. Allele similarity scores were also calculated to analyze pairwise relationships among the genotypes.

### Association analysis

Prior to association analysis, the SNPs were filtered to exclude those with greater than 20% missing data and those with a minor allele frequency (MAF) < 0.05 leaving a total of 15,229 high quality SNP markers. The remaining missing data were imputed using BEAGLE 3.3.2 (Browning and Browning, 2016). Based upon the Pearson correlation coefficient between phenotype and principle components (PC), the significantly correlated PCs were used as covariates in the final model. Fixed and random model Circulating Probability Unification (FarmCPU) (Liu et al., 2016) was used for the marker-trait association analysis, which was implemented in GAPIT2 (Tang et al., 2016). Significant associations were tested using the Bonferroni correction method (Bland and Altman, 1995) with α = 0.05, which is equivalent to the marker-wise threshold *P*-value = 3 × 10^−6^ (0.05/15,229 SNPs).

Due to the diverse backgrounds of the lines included in the association mapping panel and the large influence that the high molecular weight (HMW) glutenins can have upon soft wheat end-use quality, the panel was screened for alleles at *Glu-D1* (Liu et al., 2008) using Kompetitive Allele Specific PCR (KASP) markers (LGC Group) specific to that gene. The correlation analysis was conducted on each of the phenotypic traits to determine if the allelic diversity at the *Glu-D1* locus significantly affected each trait. The high molecular weight (HMW) glutenin data were added into the GWAS model as covariate when the trait was significantly influenced. The proportion of explained phenotypic variance by the SNP was calculated as follows:

$$R^{2}=\sum_{i=1}^{n}{\left(\hat{y_{i}}-\bar{\hat{y}}\right)}^{2}/\sum_{i=1}^{n}{\left(y_{i}-\bar{y}\right)}^{2}$$

where *y*_*i*_ is the observed phenotype value, and $\hat{y_{i}}$ is the estimated phenotype value from the multiple linear regression model that was fitted using all significant SNPs as the independent variable with fixed effect. Regression analysis was further conducted for cookie diameter to determine the amount of phenotypic variation explained by significant SNPs directly associated with these traits or other traits affecting cookie diameter. We used JMP Genomics 6.0 to compare models that included only significant markers or phenotypic data and the full model with both parameters.

## Results

### Trait statistics, heritability estimates and correlations

Significant phenotypic variation was present in this association mapping panel for the measured end-use quality traits (Table 1). Each of the traits followed a continuous distribution and the standard errors were relatively low. Narrow-sense heritability (*h*^2^) estimates ranged from 16 to 89%. High *h*^2^ traits (above 70%) included kernel hardness, break flour yield, lactic acid SRC, water SRC and cookie diameter. Low *h*^2^ traits (below 35%) included grain protein, flour protein and most of the baking parameter traits except for cookie diameter.

Significant phenotypic correlations, either positive or negative, were observed within and across trait categories (Supplementary Table 1). The highest correlation coefficient (*r* = 0.99) was observed between mixograph peak time and mixograph width. All milling traits were positively correlated to each other and ranged from *r* = 0.58–0.79. Cookie diameter was significantly correlated with all traits except test weight. Cookie diameter was positively correlated with all milling traits, and additionally flour swelling volume, and had a negative correlation with all the other traits.

**Figure 1:**
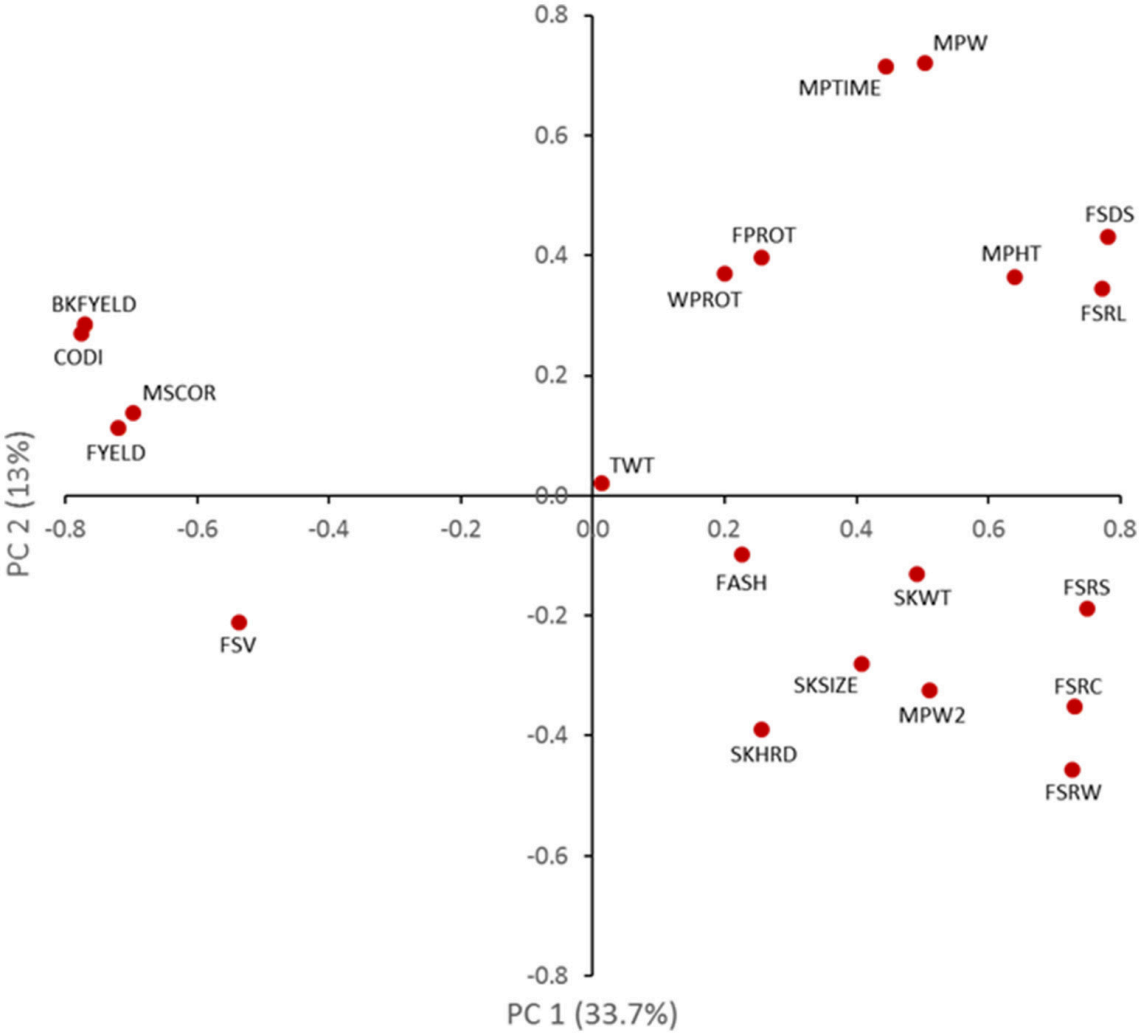
Biplot of principal components showing the phenotypic correlation of end-use quality traits in a soft white winter wheat diversity panel. SKHRD, kernel hardness; SKSIZE, kernel size; SKWT, kernel weight; TWT, test weight; WPROT, gain protein; BKFYELD, break flour yield; FYELD, total flour yield; MSCOR, milling score; FASH, flour ash; FPROT, flour protein; FSDS, flour SDS sedimentation; FSRC, carbonate solvent retention capacity; FSRL, lactic acid solvent retention capacity; FSRS, sucrose solvent retention capacity; FSRW, water solvent retention capacity; FSV, flour swelling volume; MPTIME, mixograph peak time; MPW, mixo graph height; MPW, mixograph width; MPW2, mixograph width 2 mins; CODI, cookie diameter.

The complexity of the correlation matrix for all end-use quality traits was visualized using PCA (Figure 1). The first PC axis (PC1) corresponds to the direction and degree in which correlated traits are related, either positive or negative. Cookie diameter, flour swelling volume, and all of the flour parameter traits were positively correlated and had negative values in the first PC. This group of traits was negatively correlated with grain morphology (kernel size and weight), mixograph data and SRC tests, of all which had positive PC1 values. Grain morphology, mixograph data and SRC tests were positively correlated. The second PC axis (PC2) further describes the affinity of different traits as they influence specific end-use qualities. Flour SDS sedimentation and lactic acid SRC both estimate gluten strength, which directly affects dough strength represented by the mixograph height data. SRC tests and mixograph height both had positive PC2 values. Flour SDS sedimentation and lactic acid SRC were highly correlated (*r* = 0.88) (Supplementary Table 1). Both traits were also correlated with mixograph height (*r* = 0.61 with lactic acid SRC and *r* = 0.49 with flour SDS). On the other hand, sucrose, carbonate and water SRC had negative PC2 values. Carbonate and sucrose SRC relate to the level of starch damage and arabinoxylan content, respectively. Water SRC estimates global water absorption, with higher values indicating among other things, more starch damage. These traits had positive correlations, but carbonate and water SRC were the most highly correlated (*r* = 0.78).

### Marker statistics, LD and population structure

**Figure 2:**
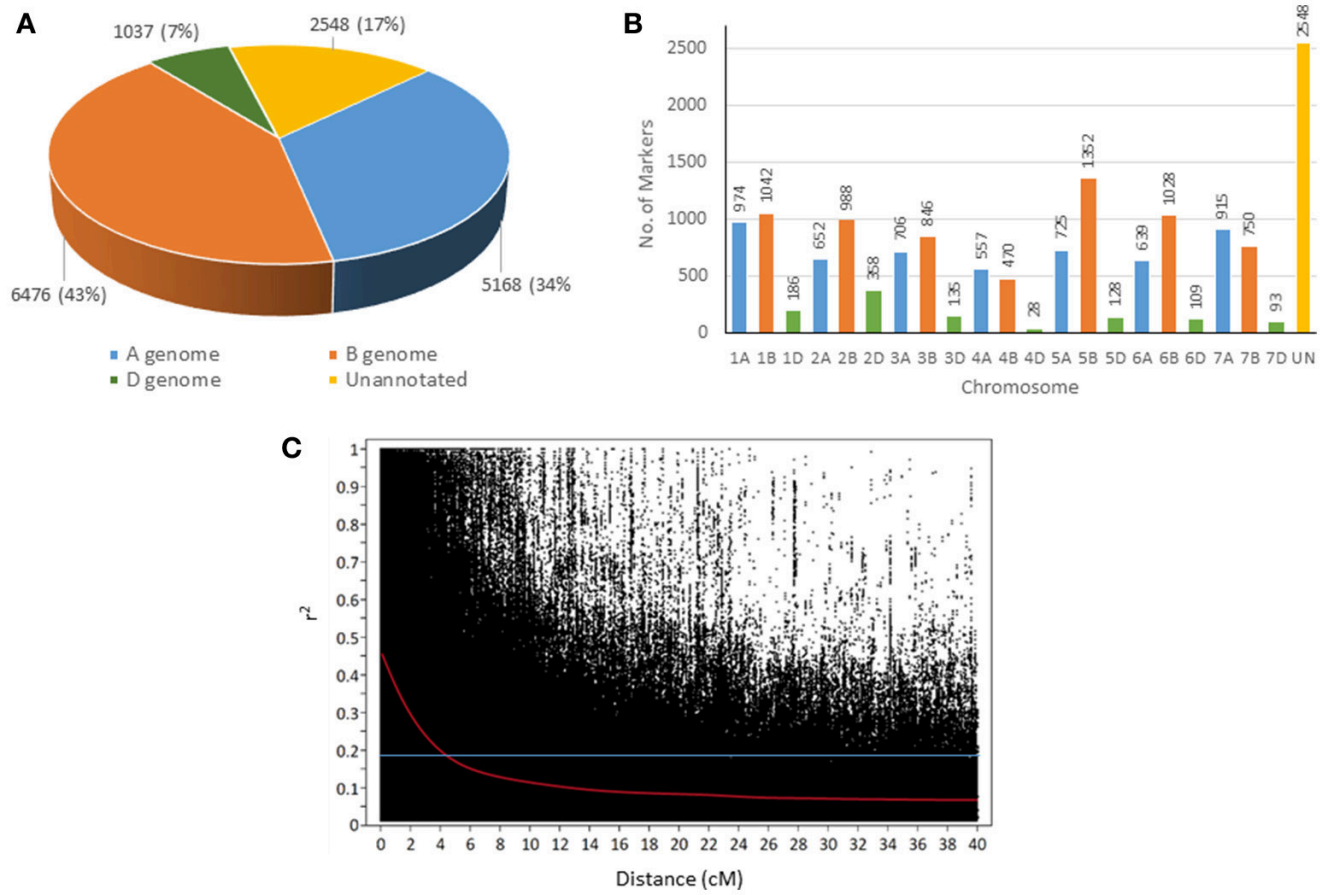
Distribution of 15,229 SNP markers across the genome **(A)** and different chromosomes **(B)**. A scatterplot **(C)** showing the decline of genome-wide linkage disequilibrium (LD) r^2^ over genetic distances (cM). The horizontal line corresponds to the 95th percentile of distributions of unlinked (>40 cM) markers at *r*^2^ = 0.18.

A total of 15,229 polymorphic SNP markers were tested for association with end-use quality traits. About 84% of the markers were annotated using a consensus map (Wang et al., 2014) whereas 17% did not yet have a genomic position on the consensus map (Figures 2A,B). The B genome had the highest number of markers (6,476) followed by the A genome with 5,168 markers. The D genome was the least covered with only 1,037 markers. Among all chromosomes, 5B had the most markers (1,1352) and 4D the fewest with 28 markers. Average marker density was 1 SNP every 0.44 cM with the most-dense marker coverage in chromosome 5B at 1 SNP every 0.06 cM (Supplementary Table 2). A large proportion (73%) of the genotypes carried the Dx2 + Dy12 subunit combination (*Glu-D1* 2+12) compared to 16% with the Dx5 + Dy10 subunit (Supplementary Figure 1).

Linkage disequilibrium was calculated for locus pairs within the same chromosomes. About 56% of intra-chromosomal pairs had significant LD (*P* < 0.01), 17% of which had *r*^2^ > 0.2. Genome-wide LD decay was observed using a scatterplot of LD (*r*^2^) plotted against inter-marker distance (cM) (Figure 2C). LDs with *r*^2^ > 0.2 extended to distances up to 10 cM. Rapid LD decay was observed from *r*^2^ = 0.6 of locus pairs with 0 cM distance to *r*^2^ = 0.26 within 5 cM of genetic distance across all chromosomes.

**Figure 3:**
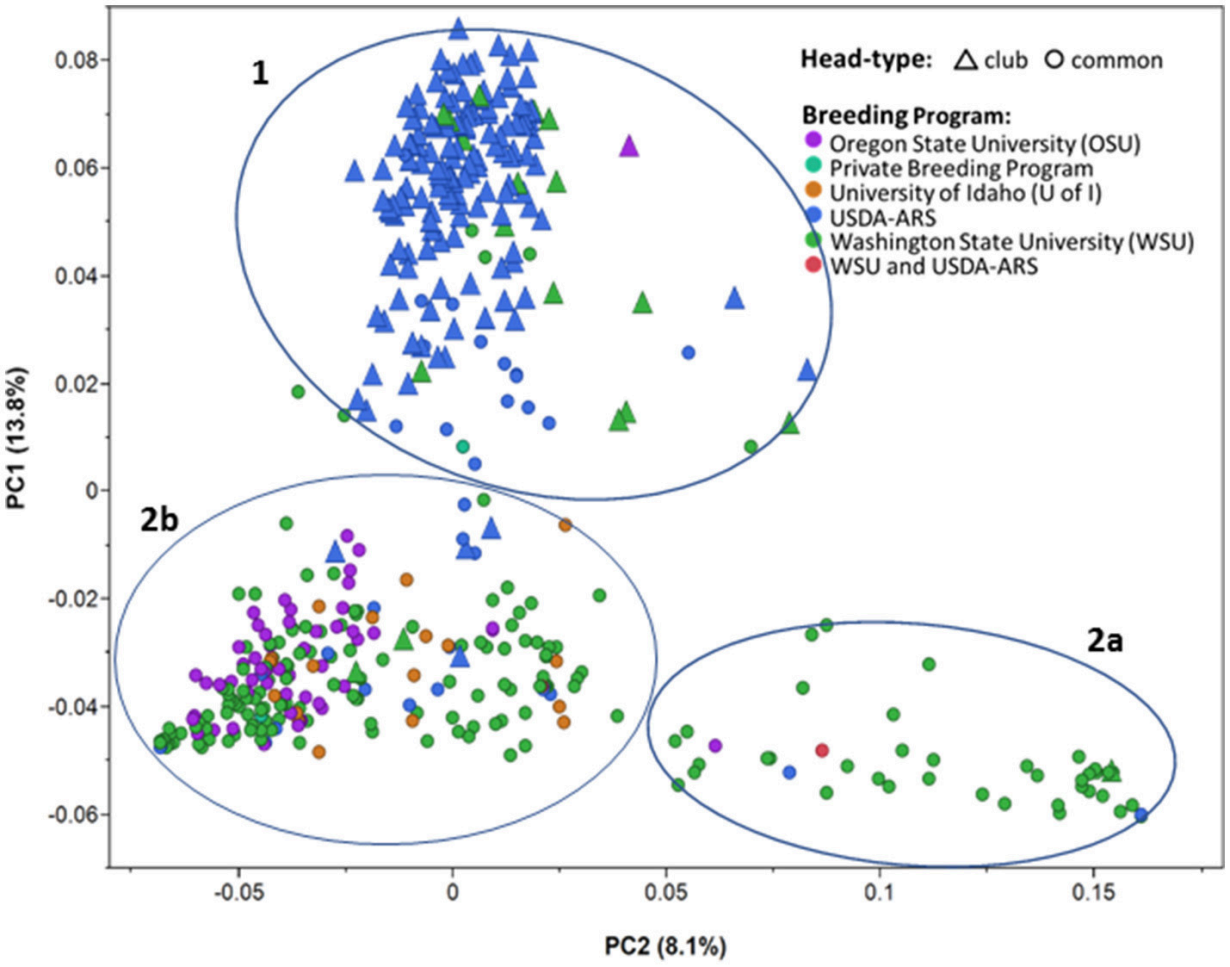
Principal component analysis (PCA) of a soft white winter wheat diversity panel using 15,229 SNP markers from all genomes showing two major sub-populations representing head type: club (1) or lax from (2) wheat breeding programs in the PNW. The lax form group can be further subdivided into two groups **(2a,b)**. The phenotypic variation explained by each PC axis are as follows: PC1 = 13.8% and PC2 = 8.1%.

Prior knowledge about the association mapping panel indicated the presence of at least two sub-populations that represent different wheat market sub-classes which are differentiated by head type: lax or club (Naruoka et al., 2015). This feature was further supported by principle component analysis showing two major subpopulations defined by the first principal component (Figure 3). Even though there was some overlap between the two sub-populations, there was a clear distinction between club (37%) and lax (64%) head types. The club wheat genotypes predominately originated from the USDA-ARS wheat breeding program based in Pullman, WA, whereas the lax wheat genotypes came from the Washington State University, University of Idaho, Oregon State University, and other private breeding programs. In PC2, a smaller subgroup within the lax subpopulation was also observed. Based on pedigree information, the genotypes in this subgroup had at least 50% of their genetic background derived a common variety “Eltan”, a widely accepted variety in the PNW in the late 1990's. This was further validated by their high allele similarity scores (>50%) with “Eltan.” Overall population F_ST_ (0.31) showed a substantial genetic differentiation between subpopulations, which was also consistent with their genetic distances (Supplementary Table 2).

### Detection of marker-trait associations (MTAs) for end-use quality traits

**Figure 4:**
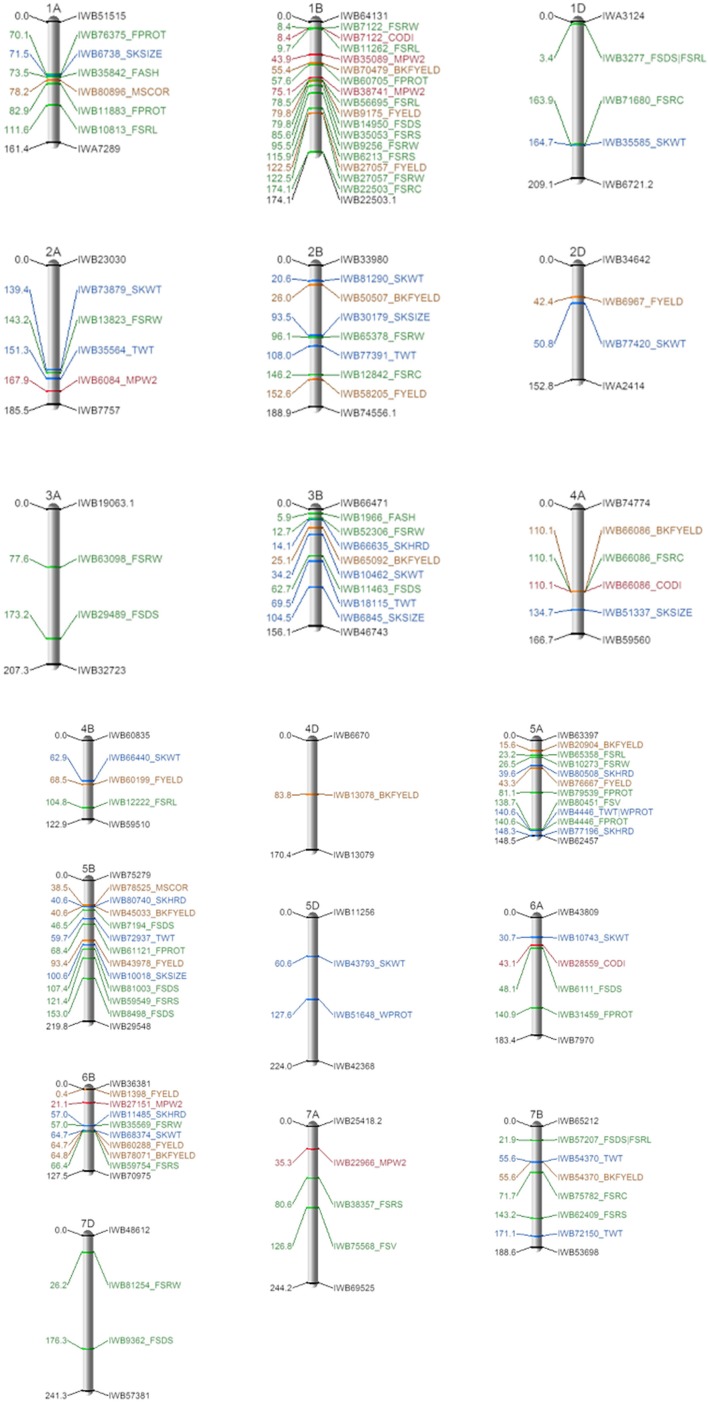
Location of the 105 MTAs for end-use quality traits detected in the Pacific Northwest soft white winter wheat diversity panel. Genetic linkage maps of the chromosomes are based on the wheat consensus SNP map (Wang et al., 2014). Values on the left are genetic distance in centimorgan (cM). On the right, SNP ID and the associated end-used quality traits. See Table 1 for complete description of trait abbreviations. Trait groupings are color coded. Blue, grain characteristics; Green, flour parameters; Brown, milling traits; Red, baking parameters. SKHRD, kernel hardness; SKSIZE, kernel size; SKWTkernel weight; TWT, test weight; WPROT, grain protein; BKFYELD, break flour yield; FYELO, total flour yield; MSCOR, milling score; FASH, flour ash; FPROT, flour protein; FSOS, flour SDS sedimentation; FSRC, carbonate solvent retention capacity; FSRL, lactic acid solvent retention capacity; FSRS, sucrose solvent retention capacity; FSRW, water solvent retention capacity; FSV, flour swelling volume; MPW2, mixograph width 2 mins; CODI, cookie diameter.

The mixed linear model used in the association analysis varied among different traits by the number of principal components and *Glu-D1* as an additional covariate (Supplementary Table 3). Taking all traits together, a total of 105 MTAs was significant across 19 wheat chromosomes (Figure 4). Summary information for each MTA can be found in Supplementary Table 4. Sixty MTA were detected on the B genome, followed by A (34) and D (11) genomes. Chromosome 1B was the most informative with 16 MTAs, whereas chromosome 4D was the least informative having only one associated marker. An average of five MTAs per trait were observed. The highest number of MTAs per trait were for flour SDS sedimentation and water absorption with 10 SNP markers each. Traits with the fewest number of associated markers include: flour ash (2), flour swelling volume (2), milling score (2) and grain protein (2). There were no significant associations detected for mixograph height, width and peak time in this panel. Among the 105 MTAs, 49 had *R*^2^ values between 5 – 44% (Table 2); they were widely distributed throughout the genomes except on chromosomes 3D, 4D, 6D, and 7D. More information on the 49 MTAs will be presented in the succeeding sections.

**Table 2:** Markers associated with end-use quality traits in the Pacific Northwest soft white winter wheat diversity panel.

**Trait**	**SNP ID^b^**	**SNP name^c^**	**Chrom^d^**	**Pos (cM)^e^**	***P-value*^f^**	**Alleles^g^**	**MAF^h^**	**α^i^**	***R^2^*^j^**
**GRAIN CHARACTERISTICS**									
SKHRD^a^	IWB80508	wsnp_Ku_c4389_7970859	5A	39.62	7.68E-09	C/T	0.07	+	0.06
SKSIZE	IWB30179	Excalibur_rep_c106124_239	2B	93.47	5.48E-07	A/G	0.36	+	0.07
	IWB51337	Ra_c1897_2401	4A	134.66	1.44E-06	G/A	0.17	–	0.09
SKWT	IWB73879	Tdurum_contig93508_295	2A	139.35	1.74E-06	A/G	0.29	–	0.19
	IWB77420	wsnp_Ex_c25311_34578436	2D	50.83	7.11E-10	C/T	0.37	–	0.27
	IWB10462	BS00070455_51	3B	34.20	2.67E-06	G/T	0.39	–	0.10
	IWB66440	Tdurum_contig10300_400	4B	62.92	1.55E-07	A/C	0.31	–	0.07
	IWB43793	Kukri_c29969_543	5D	60.61	2.27E-06	G/T	0.45	+	0.06
	IWB68374	Tdurum_contig15235_951	6B	64.71	1.87E-06	G/A	0.42	–	0.21
TWT	IWB77391	wsnp_Ex_c24711_33964543	2B	108.04	2.57E-08	C/A	0.21	+	0.09
	IWB18115	D_GB5Y7FA02I44NU_340	3B	69.53	1.46E-06	C/T	0.11	–	0.09
	IWB4446	BobWhite_c8266_227	5A	140.59	2.14E-09	C/A	0.20	–	0.07
WPROT	IWB4446	BobWhite_c8266_227	5A	140.59	9.66E-07	C/A	0.20	–	0.06
**MILLING TRAITS**									
BKFYELD	IWB66086	TA006348-0950	4A	110.13	7.53E-07	C/T	0.08	–	0.11
	IWB45033	Kukri_c40388_844	5B	40.57	4.94E-07	G/A	0.31	+	0.09
	IWB54370	RAC875_c16839_188	7B	55.64	4.39E-08	G/A	0.45	–	0.05
FYELD	IWB9175	BS00064032_51	1B	79.77	2.37E-07	A/G	0.35	–	0.20
	IWB27057	Excalibur_c49496_705	1B	122.52	5.04E-15	C/T	0.22	–	0.23
	IWB6967	BS00022211_51	2D	42.37	1.05E-06	A/C	0.43	–	0.08
	IWB60199	RAC875_c6865_349	4B	68.45	4.93E-07	G/A	0.42	+	0.27
	IWB76667	wsnp_Ex_c14812_22928900	5A	43.27	3.72E-10	T/C	0.33	+	0.20
MSCOR	IWB78525	wsnp_Ex_c5915_10379277	5B	38.50	3.10E-08	A/G	0.35	–	0.06
**FLOUR PARAMETERS**									
FASH	IWB35842	IACX219	1A	73.48	1.14E-06	G/T	0.06	–	0.05
FPROT	IWB4446	BobWhite_c8266_227	5A	140.59	2.07E-08	C/A	0.20	–	0.07
	IWB31459	Excalibur_rep_c98042_438	6A	140.87	3.24E-09	T/C	0.21	+	0.05
FSDS	IWB14950	CAP8_c818_370	1B	79.77	1.35E-11	G/T	0.31	+	0.24
	IWB3277	BobWhite_c4303_524	1D	3.40	1.77E-08	T/C	0.36	–	0.48
	IWB29489	Excalibur_c94962_57	3A	173.15	2.06E-06	A/G	0.24	+	0.24
	IWB6111	BS00009782_51	6A	48.09	2.74E-11	T/C	0.30	–	0.05
	IWB57207	RAC875_c36670_72	7B	21.86	1.47E-09	C/A	0.08	+	0.20
FSRC	IWB66086	TA006348-0950	4A	110.13	1.44E-06	C/T	0.08	+	0.08
FSRL	IWB10813	BS00075532_51	1A	111.56	6.79E-09	G/A	0.07	+	0.07
	IWB56695	RAC875_c32077_284	1B	78.45	1.40E-08	C/T	0.34	+	0.30
	IWB3277	BobWhite_c4303_524	1D	3.40	7.10E-07	T/C	0.36	–	0.44
	IWB12222	BS00104279_51	4B	104.79	3.85E-07	T/C	0.23	+	0.12
	IWB57207	RAC875_c36670_72	7B	21.86	2.89E-06	C/A	0.08	+	0.24
FSRS	IWB35053	IAAV5588	1B	85.57	4.68E-10	C/A	0.31	+	0.05
	IWB59549	RAC875_c60758_585	5B	121.43	3.78E-08	G/T	0.11	+	0.06
	IWB38357	Ku_c12886_1250	7A	80.64	2.24E-06	A/G	0.23	+	0.12
FSRW	IWB7122	BS00022504_51	1B	8.36	1.33E-11	G/A	0.06	+	0.09
	IWB9256	BS00064349_51	1B	95.49	1.40E-06	T/C	0.33	-	0.15
	IWB27057	Excalibur_c49496_705	1B	122.52	1.35E-06	C/T	0.22	+	0.08
	IWB65378	TA001322-1176	2B	96.14	1.10E-09	A/G	0.47	-	0.05
	IWB10273	BS00068257_51	5A	26.50	3.34E-08	C/T	0.25	-	0.27
FSV	IWB80451	wsnp_Ku_c38543_47157828	5A	138.73	1.74E-08	C/T	0.22	+	0.20
**BAKING PARAMETERS**									
MPW2	IWB6084	BS00009594_51	2A	167.87	1.95E-07	T/C	0.46	-	0.07
	IWB35089	IAAV5782	1B	43.86	1.41E-09	G/A	0.39	-	0.22
CODI	IWB7122	BS00022504_51	1B	8.36	5.87E-07	G/A	0.06	-	0.12
	IWB66086	TA006348-0950	4A	110.13	7.47E-07	C/T	0.08	-	0.08

a *SKHRD, kernel hardness; SKSIZE, kernel size; SKWT, kernel weight; TWT, test weight; WPROT, grain protein; BKFYELD, break flour yield; FYELD, total flour yield; MSCOR, milling score; FASH, flour ash; FPROT, flour protein; FSDS, flour SDS sedimentation; FSRC, carbonate solvent retention capacity; FSRL, lactic acid solvent retention capacity; FSRS, sucrose solvent retention capacity; FSRW, water solvent retention capacity; FSV, flour swelling volume; MPW2, mixograph width 2 mins; CODI, cookie diameter*.

b, c, d, e *SNP ID, SNP name, chromosome location and position based on wheat 90K consensus map (Wang et al., 2014)*.

f *Nominal p-values*.

g *Alleles for specific SNP markers. Underlined nucleotides represent minor alleles*.

h *Minor allele frequency (MAF). Frequency of minor allele in the panel*.

j *Alpha (α) denotes the allelic effect of the minor alleles*.

i *Phenotypic variation explained by the SNP*.

### Distribution and co-localization of 49 MTAs

Thirteen MTAs for grain characteristics were detected across nine chromosomes (Table 2 and Supplementary Table 5). Six MTAs that explained between 6 – 27% of the phenotypic variation in single kernel weight were identified on 2A, 2D, 3B, 4B, 5D, and 6B. *IWB77420* on chromosome 2D was the most significant MTA with *R*^2^ = 0.27. The allele for heavier kernel weight was present in 99% in the genotypes with the lax head type, but only carried by 1% of the club wheats. Three MTAs for test weight on chromosomes 2B (*IWB77391*), 3B (*IWB18115*) and 5A (*IWB4446*) explained up to 9% of the phenotypic variation. More than 80% of the genotypes carried the alleles for higher test weight in *IWB18115* and *IWB4446*, whereas, only 21% had the favorable allele of *IWB77391* (Supplementary Table 5). *IWB4446* was also associated with grain protein and flour protein, explaining 6 and 7% of the phenotypic variation, but the allelic effect was positively correlated, which is not desired in soft white wheat. Two MTAs for single kernel size (diameter) were observed in chromosomes 2B (*IWB30179*) and 4A (*IWB51337*), together accounting for 16% of the trait variation. An MTA that explained 6% of the variation in kernel hardness was detected in chromosome 5A (*IWB80508*). The majority of both the club and lax head-type sub-groups (92 and 93%, respectively) carried the allele for softer kernel.

Nine markers on chromosomes 1B, 2D, 4A, 4B, 5A, 5B, and 7B were associated with break flour yield (3), flour yield (5) and milling score (1) (Table 2). The favorable alleles for these MTAs were highly represented in the club head genotypes compared to lax head-type genotypes (Supplementary Table 5). *IWB66086* at 110.1 cM on 4A accounted for most of the variation (11%) in break flour yield as well as 8% of the variation in carbonate SRC and in cookie diameter. Other markers associated with break flour yield included *IWB45033* (*R*^2^ = 0.09) at 40.6 cM on 5B and *IWB54370* (*R*^2^ = 0.05) at 55.6 cM on 7B. *IWB78525*, an MTA for milling score on 5B, was 2 cM from the break flour yield MTA. Four out of five MTA for higher flour yield had *R*^2^ values ≥ 20%, and two of these, *IWB9175* and *IWB27057*, were positioned in the short and long arms of chromosome 1B, respectively. Aside from flour yield, *IWB27057* was also strongly associated with water SRC (*R*^2^ = 0.22). The strongest MTA (*IWB60199*) for flour yield was positioned at 68 cM on 4B and the allele for higher flour yield was highly represented (84%) in the club wheats (Supplementary Table 5).

The number of marker-trait associations for flour quality traits were greatest for flour SDS sedimentation, lactic acid SRC and water SRC, with 5 SNP markers each (Table 2). *IWB3277* (1D) and *IWB57207* (7B) were both associated with flour SDS sedimentation and lactic acid SRC. *IWB3277*, on the short arm of chromosome 1D, accounted for at least 44% of the phenotypic variation in these traits and the alleles for lower values were present in 74% of the genotypes (Supplementary Table 5). However, *IWB14950* (79.8 cM) and *IWB56695* (78.5 cM) on chromosome 1B were also associated with flour and lactic acid SRC, respectively. The MTA for water SRC with the greatest effect (*R*^2^ = 0.27) was positioned at 26.5 cM on chromosome 5A (*IWB10273*). Water SRC and cookie diameter shared a locus on chromosome 1B at 8.4 cM (*IWB7122*) explaining 9-12% of the phenotypic variation. The favorable allele for flour ash (lower ash content is preferred) on chromosome 1A (*IWB35825*) was present in only 6% of the genotypes, mostly coming from the Oregon State University wheat breeding program (Supplementary Table 5). An MTA was identified for flour protein on chromosome 6A (*IWB31459*) and the allele associated with lower protein was present in 79% of the genotypes. A large effect flour protein MTA was positioned at 138 cM on chromosome 5A and explained 20% of the phenotypic variation. The minor alleles of the four MTAs for

baking parameters contributed to reduced peak width and cookie diameter. Of these, the MTA for mixograph peak width 2 mins, *IWB35089* (1B), had the largest effect *R*^2^ = 0.22 (Table 2).

## Discussion

### Variation and heritability among end-use quality traits

The use of historical data facilitated a larger number of phenotypic observations (e.g., as many as 5,000 observations for test weight) to capture a wider range of performance in end-use quality traits that represented past and current soft winter wheat germplasm in the Pacific Northwest. The use of BLUPs accommodated departures from normality, while at the same time accounting for year and environmental effects that are common in unbalanced multi-location trials (Smith et al., 2005). Heritability estimated using genome-wide markers showed a clear picture of the proportion of additive variance for each trait. Quality traits with high heritability such as kernel hardness, break flour yield, lactic acid SRC, water SRC, and cookie diameter, indicate that a large portion of their expression is genetically controlled and that breeding lines can be selected for substantial genetic gains in the end-use quality of soft winter wheat (Guttieri et al., 2001). High heritability estimates for these traits were also reported in soft winter wheat using biparental populations (Carter et al., 2012; Jernigan et al., 2017) and wheat germplasm core collections (Tadesse et al., 2015). In this study, traits with lower heritability (like grain protein, flour protein and some baking parameters) also had the fewest associations and their MTA had minor effects. These results are consistent with the complex and polygenic nature of quality traits (Turner et al., 2004).

### MTA for grain characteristics

The main goal of this study was to dissect the underlying genetics controlling end-use quality traits of soft wheat, a class of wheat that is different from the hard wheat preferred for baking bread (Kiszonas and Morris, 2018). Soft and hard wheats are mainly classified by the *Ha* locus and the puroindoline genes, *Pina* and *Pinb* in the distal portion of chromosome 5DS (Sourdille et al., 1996; Morris, 2002). The hardness locus has a strong influence on most end-use quality traits such as kernel texture, flour yield, flour particle size, starch damage, and dough strength (Campbell et al., 2001; Nelson et al., 2006; Boehm Jr et al., 2017). No marker on chromosome 5DS was associated kernel hardness, but universally, the *Ha* locus is fixed for the wild type soft allele in our panel. We detected a marker (*IWB80508*) for single kernel hardness on chromosome 5A. A similar locus in the same region on chromosome 5A, identified by *wPt-1165*, was also detected by Bordes et al. (2011) in a global core collection of 372 wheat accessions further supporting our hypothesis on the importance of this region in controlling kernel hardness. The MTA in 5BL (*IWB80740*) was < 1 cM on the consensus map to a kernel hardness QTL (*QHa.wak*) identified in a bi-parental population derived from a cross between the club head cultivar “Coda” and the lax head cultivar “Brundage” (Jernigan et al., 2017). Both Coda and Brundage were included in our panel, validating this result. Softer kernels often have higher milling performance and flour yields (Carter et al., 2012) primarily due to the differences in fracture patterns caused by milling. Soft wheats endosperm tends to fracture through cells, vs. at the cell wall in hard wheats, leading to smaller particle size distribution, less starch damage, and lower water absorption (Hoseney et al., 1988). Not surprisingly, we detected an MTA (*IWB76667*) for flour yield near (~4 cM) *IWB80508* on chromosome 5A which explained about 20% of the phenotypic variation. In addition, co-localized MTAs for single kernel hardness and water absorption SRC were also detected at 57 cM on chromosome 6BS.

Kernel weight, kernel size, grain protein, and test weight are also important factors influencing wheat milling performance (Morris and Rose, 1996; Campbell et al., 1999). Kernel size and weight were highly correlated in this study; however, we did not detect any markers associated with both traits. It is possible that no major gene or MTA is controlling both traits and different genomic regions with minor effects contribute to the overall trait expression. The kernel weight MTA (*IWB68374*) was also reported in Sun et al. (2008) as a pleiotropic region controlling starch quality. To date, the most phenotypic variation explained by a single QTL (*IWB77420*) for kernel weight and size is only 28% (Jernigan et al., 2017) identified from wheat with a lax head type. *IWB77420* was also associated with single kernel weight in this study and the allele for heavier kernel was present in 99% of the genotypes with lax head type and only 1% of the genotypes with club head types. This MTA is linked to the *compactum* (*C*) locus for club head type. *IWB77420* was 2 cM proximal to the flanking marker of the *C* locus, *wmc144* (Johnson et al., 2008), in the integrated map (Maccaferri et al., 2015). Club wheat has unique spike morphology, due to the *C* locus on chromosome 2DL (Johnson et al., 2008) that results in redistribution of yield components. The *C* locus directly influences seed size, seed number, and test weight (Gul and Allan, 1972). Club cultivars have smaller seeds than lax cultivars, but spikelet fertility and number of seeds per spike is greater in club wheat (Zwer et al., 1995).

Grain protein is an essential component affecting flour functionality. Unlike hard wheats, lower protein levels are desired for soft wheats to minimize gluten formation and mixing strength that is otherwise needed in bread doughs (Souza et al., 2012). The positive correlation between protein concentration and flour SDS sedimentation (a measure of gluten strength) provides further evidence of their direct relationship. All MTAs for protein concentration explained no more than 7% of the phenotypic variation. Grain and flour protein concentrations have low heritability and are largely dependent on the environment (Turner et al., 2004). Grain and flour protein were highly correlated. A marker (*IWB4446*) on 5AL was associated with both traits and is close (less than 3 cM) to a previously reported flour QTL (*QFpro.wak*) (Jernigan et al., 2017). *IWB4446* was also associated with test weight despite having a low correlation with protein concentration. This MTA can be useful in hard wheats as selection for higher test weight would lead to an increase in protein concentration. However, this strategy would not be beneficial for soft wheats in maintaining lower protein values. If *IWB4446* will be used to select for lower protein concentration, reduction in test weight should be compensated by using MTA from other chromosomes. Test weight is of commercial value to wheat growers and should be given equal consideration with the other quality traits.

### MTA for milling traits

Milling performance directly translates into greater profit margins for flour millers. Hence, breeding programs aim for soft wheats with high break flour yield, flour yield, and milling score. The moderate to high heritability estimates and positive correlations among milling traits suggest simultaneous progress from selection can be achieved. Selecting alleles that increase these traits could lead to higher milling scores (Supplementary Table 6). Club wheat varieties like “ARS-Crescent,” “Cara,” and “Chukar” that are considered to have “excellent” quality carried all 9 favorable alleles for superior milling traits compared to “Xerpha,” a common (lax head) wheat graded as a “least desirable” variety for Washington, Northern Idaho and Oregon, which only had two favorable alleles (Washington Grain Commission, 2017). Soft winter wheat varieties (with lax head type) like ARS-Amber, BrundageCF and Jasper carried at least five favorable alleles of the nine QTL for milling traits were also highly preferred in the PNW. MTAs for break flour yield (*IWB45033*) and milling score (*IWB78525*) were identified less than 2 cM apart in chromosome 5B. A QTL for kernel hardness (*QHa.wak*) was also reported in this region and contributed to variation in break flour yield (Jernigan et al., 2017). *IWB60199* on chromosome 4B (68.4 cM), which explained 27% of the variation in flour yield, was represented in 17 and 84% of the genotypes with lax and club head types, respectively (Table 2 and Supplementary Table 6). Even though this marker is near a major gene for plant height (*Rht-B1*) on 4B, we do not think there is a pleiotropic effect of dwarfing genes on flour yield. The majority (85%) of lines with lax head type carry the semi-dwarf allele *Rht-B1b*, mainly because this allele has historically been used in combination with selection for better emergence potential in lower rainfall regions of the Pacific Northwest. The club wheat lines, which have been bred and selected for higher flour yield, have a higher predominance of the semi-dwarf allele *Rht*-*D1b*, mainly because they have not undergone the intense selection for better emergence as the lines with lax heads have. Thus, although there is a correlation between dwarfing genes and flour yield, we do not believe it is causal.

To our knowledge, the two large effect MTAs for total flour yield on the short and long arms of chromosome 1B have not been reported, especially *IWB27057*, which was also associated with lower water absorption SRC. *IWB27057* is 15 cM proximal from the high molecular weight glutenin gene (*Glu-B1*) in chromosome 1B. Soft wheats with lower water absorption are important in baking cookies. Upon further validation, these MTAs can be used to select soft wheats with improved total milling performance.

### MTA for flour parameters

The strength of correlation among pairs of SRC traits were consistent with the clustering of MTA in certain genomic regions such as flour SDS sedimentation and lactic acid SRC (*r* = 0.87), which are both predictors of gluten strength. MTAs for flour SDS sedimentation and lactic acid SRC were detected on chromosomes 1BS and 1DS, respectively, near known glutenin genes *Glu-B1* and *Glu-D3*, respectively (Zheng et al., 2009). These MTAs are linked with major glutenin genes. The high frequency of the favorable allele on 1DS among club lines can be beneficial in breeding for wheats with lower gluten content/strength. The MTA (*IWB9175*) for total flour yield which was also detected in this region was consistent with the significant correlations among these traits. No additional MTAs were detected in the region of *Glu-D1* because we included variation at *Glu-D1* as a covariate in the association analysis. The MTA (*IWB57207*) on chromosome 7BS is potentially a novel locus controlling flour SDS sedimentation and lactic acid SRC. The high frequency of this MTA in this panel makes it a valuable resource for introducing new alleles with diverse genetic backgrounds.

Water SRC co-localized with other quality traits on 1BS and 1BL, and with cookie diameter and total flour yield. Higher water SRC is partly a consequence of starch damage from milling and non-starch polysaccharides, including arabinoxylans (Guttieri et al., 2008; Souza et al., 2012; Kiszonas et al., 2013b). A higher concentration of water-soluble arabinoxylans will result in smaller (less desirable) cookie diameters (Guttieri et al., 2001, 2008; Ramseyer et al., 2011). Arabinoxylan content in wheat flour is measured by the sucrose SRC test, thus, it was not surprising to also detect a marker for sucrose SRC near the water absorption SRC marker on 1BL and 6BS (Figure 4). Partly due to low heritability, only two MTAs each were detected for flour ash and flour swelling volume. The region (*IWB35842*), associated with flour ash on chromosome 1A, was likely the same as the QTL for flour ash identified from a double haploid mapping population (El-Feki et al., 2013). Lower flour ash is desired especially in export markets because higher ash flours are associated with reduced flour functionality affecting most batters and dough (Liu et al., 2011). The high representation of the allele for lower ash content in this population indicates that breeding programs in the Pacific Northwest have indirectly selected for this allele by phenotypically selecting for high flour yield, high milling score, and low flour ash. The marker (*IWB80451*) associated with flour swelling volume on 5A is potentially an important locus due to its large effect and availability especially in genotypes with lax head type.

### Implications for improvement of cookie diameter

The sugar snap cookie test is considered the best single indicator of the overall quality of soft wheat flour (Morris and Rose, 1996; Miller et al., 1997) and has been the primary means of selecting breeding materials. The best cookies (with greater diameter) are derived from wheats with softer kernels, higher break flour yield and total flour yield, lower ash content, lower starch damage, lower gluten strength, and lower water absorption. The modest effects (*R*^2^ = 0.8–0.12) of the MTAs for cookie diameter, however, illustrated the complexity of this trait. A combination of different MTA from the traits mentioned above would be an effective strategy to achieve substantial gains. A regression analysis was used to model cookie diameter using only phenotype data, markers, or a combination of both. There was a 7% improvement in the *R*^2^ of the model when combining marker and phenotypic data in predicting cookie diameter (Supplementary Figure 2). Kiszonas et al. (2015) also modeled cookie diameter using a similar set of traits on 120 soft wheats and reported an *R*^2^ of 0.59. Breeding lines carrying most if not all of the alleles for greater cookie diameter were identified in this study and can be used by breeders to produce soft wheat varieties with superior end-use qualities (Supplementary Table 6).

### Toward genomic selection for end-use quality traits

One advantage of performing association mapping in elite and adapted lines rather than on a diversity panel is the opportunity to directly utilize the identified alleles in regional breeding programs. Genotypes that carry the favorable alleles for different end-use quality MTAs can be cycled back into breeding programs as parental material to exploit transgressive segregation, pyramid desirable alleles, and ensure potential genetic gains in succeeding generations. KASP markers of the reported MTAs are commercially available (http://polymarker.tgac.ac.uk/), making it easier to test and determine the value of these MTAs in different breeding programs. Typically, end-use quality assessment is conducted later in the breeding cycle because these tests are time consuming and require a large amount of grain. MTAs with larger effects can potentially be used in marker-assisted selection to increase selection efficiency by reducing the time and cost in screening a larger number of plants. But for markers with minor effects, MAS would be inferior to phenotypic selection.

Genomic selection is a breeding method used to predict the breeding value of individual genotypes using genome-wide markers (Bernardo, 1994; Meuwissen et al., 2001). This method can simultaneously model all additive genetic variance that is unaccounted for in GWAS with complex and low heritability traits. Once training models are developed, genomic selection for end-use quality can be implemented in earlier stages of the breeding pipeline. We are currently testing different genomic selection models to improve prediction accuracy for end-use quality traits. Preliminary results indicate that a multi-trait genomic selection approach using correlated end-use quality traits significantly improved prediction accuracy.

## Author contributions

KJ: Analyzed the phenotypic data; JG: Analyzed the phenotypic and genotypic data and drafted the manuscript; MH: Performed GWAS; YZ and ZZ: Provided expert guidance in performing GWAS; CM, KG-C, ZZ, and AC: Edited the manuscript; CM and AC: Obtained funding for the project.

### Conflict of interest statement

The authors declare that the research was conducted in the absence of any commercial or financial relationships that could be construed as a potential conflict of interest.
