# Supplementary material for: Genetic Dissection of End-Use Quality Traits in Adapted Soft White Winter Wheat
Source: Front Plant Sci. 2018 Mar 9;9:271. doi: 10.3389/fpls.2018.00271 (PMC5861628; doi:10.3389/fpls.2018.00271)
Supplement: Supplementary file 1 [file Table1.docx]

Supplementary Table 1. Pearson correlation (*r*) between end-use quality traits collected from the Pacific Northwest soft white winter wheat diversity panel.

|  | **Trait^a^** | **SKHRD** |  |  |  |  |  |  |  |  |  |  |  |  |  |  |  |  |  |  |
| --- | --- | --- | --- | --- | --- | --- | --- | --- | --- | --- | --- | --- | --- | --- | --- | --- | --- | --- | --- | --- |
| **Grain** | **SKSIZE** |  | **SKSIZE** |  |  |  |  |  |  |  |  |  |  |  |  |  |  |  |  |  |
|  | **SKWT** | **-0.19** | **0.87** | **SKWT** |  |  |  |  |  |  |  |  |  |  |  |  |  |  |  |  |
|  | **TWT** | 0.13 |  |  | **TWT** |  |  |  |  |  |  |  |  |  |  |  |  |  |  |  |
|  | **WPROT** |  | 0.1 |  | 0.12 | **WPROT** |  |  |  |  |  |  |  |  |  |  |  |  |  |  |
| **Flour** | **BKFYELD** | **-0.54** | **-0.37** | **-0.37** | -0.1 | -0.1 | **BKFYELD** |  |  |  |  |  |  |  |  |  |  |  |  |  |
|  | **FYELD** |  | -0.14 | **-0.21** |  |  | **0.66** | **FYELD** |  |  |  |  |  |  |  |  |  |  |  |  |
|  | **MSCOR** | -0.1 | **-0.17** | **-0.26** | **0.16** |  | **0.58** | **0.79** | **MSCOR** |  |  |  |  |  |  |  |  |  |  |  |
| **Milling** | **FASH** |  | 0.1 | 0.15 | **-0.28** |  | -0.11 |  | **-0.63** | **FASH** |  |  |  |  |  |  |  |  |  |  |
|  | **FPROT** |  |  |  | 0.13 | **0.86** | **-0.17** |  |  |  | **FPROT** |  |  |  |  |  |  |  |  |  |
|  | **FSDS** |  | 0.14 | **0.29** |  | **0.24** | **-0.44** | **-0.52** | **-0.46** |  | **0.31** | **FSDS** |  |  |  |  |  |  |  |  |
|  | **FSRC** | **0.23** | **0.26** | **0.29** |  |  | **-0.56** | **-0.53** | **-0.53** | **0.21** |  | **0.41** | **FSRC** |  |  |  |  |  |  |  |
|  | **FSRL** |  | **0.13** | **0.28** |  |  | **-0.39** | **-0.52** | **-0.41** |  | 0.13 | **0.88** | **0.48** | **FSRL** |  |  |  |  |  |  |
|  | **FSRS** | 0.11 | **0.32** | **0.37** |  |  | **-0.52** | **-0.54** | **-0.52** | **0.18** |  | **0.49** | **0.68** | **0.5** | **FSRS** |  |  |  |  |  |
|  | **FSRW** | **0.52** | **0.31** | **0.28** |  |  | **-0.67** | **-0.49** | **-0.41** |  |  | **0.35** | **0.73** | **0.42** | **0.66** | **FSRW** |  |  |  |  |
|  | **FSV** | **-0.19** | **-0.16** | **-0.23** |  | **-0.18** | **0.3** | **0.24** | **0.26** | -0.12 | **-0.18** | **-0.44** | **-0.32** | **-0.45** | **-0.25** | **-0.31** | **FSV** |  |  |  |
| **Baking** | **CODI** | **-0.45** | **-0.41** | **-0.4** |  | **-0.18** | **0.65** | **0.44** | **0.44** | **-0.16** | **-0.21** | **-0.45** | **-0.66** | **-0.47** | **-0.58** | **-0.72** | **0.34** | **CODI** |  |  |
|  | **MPTIME** | -0.13 |  | 0.13 |  |  | **-0.16** | **-0.24** | **-0.21** |  | 0.1 | **0.59** | 0.13 | **0.61** | **0.22** |  | **-0.31** | -0.15 | **MPTIME** |  |
|  | **MPHT** | **0.19** | **0.17** | **0.24** | 0.12 | **0.39** | **-0.46** | **-0.33** | **-0.36** | **0.17** | **0.45** | **0.61** | **0.26** | **0.49** | **0.38** | **0.28** | **-0.5** | **-0.43** | **0.36** | **MPHT** |
|  | **MPW** | -0.09 |  | **0.15** |  |  | **-0.21** | **-0.27** | **-0.24** |  | 0.14 | **0.64** | **0.16** | **0.65** | **0.26** | 0.1 | **-0.35** | **-0.2** | **0.99** | **0.46** |
|  | **MPW2** | 0.09 | **0.22** | **0.27** |  |  | **-0.32** | **-0.41** | **-0.34** |  |  | **0.42** | **0.42** | **0.53** | **0.39** | **0.48** | **-0.22** | **-0.39** |  | 0.1 |

*^a^SKHRD* - kernel hardness, *SKSIZE* - kernel size, *SKWT* - kernel weight, *TWT* - test weight, *WPROT* - grain protein, *BKFYELD* - break flour yield, *FYELD* - total flour yield, *MSCOR* - milling score, *FASH* - flour ash, *FPROT* - flour protein, *FSDS* - flour SDS sedimentation, *FSRC* - carbonate solvent retention capacity, *FSRL* - lactic acid solvent retention capacity, *FSRS* - sucrose solvent retention capacity, *FSRW* - water solvent retention capacity, *FSV* - flour swelling volume, *MPTIME -* mixograph peak time, *MPW* - mixograph height, *MPW - mixograph width, MPW2* - mixograph width 2 mins, *CODI* - cookie diameter

Note: Only significant (*p < 0.05*) correlations are shown. Bold values indicate highly significant (*p < 0.001*) correlation coefficients. Cell colors correspond to correlation of traits that belong to the same category (grain, flour, milling and baking).
