# Supplementary material for: Genetic Dissection of End-Use Quality Traits in Adapted Soft White Winter Wheat
Source: Front Plant Sci. 2018 Mar 9;9:271. doi: 10.3389/fpls.2018.00271 (PMC5861628; doi:10.3389/fpls.2018.00271)
Supplement: Supplementary file 2 [file Table2.docx]

Supplementary Table 2. Dissimilarity matrix between the subpopulations identified using principal component analysis (PCA).

| Subpopulation | 1 (Club) | 2a (Lax) | 2b (Lax) |
| --- | --- | --- | --- |
| 1 (Club) | 0.00 | 0.44 | 0.28 |
| 2a (Lax) | 0.44 | 0.00 | 0.32 |
| 2b (Lax) | 0.28 | 0.32 | 0.00 |
| Genome | Mean F_ST_ |  |  |
| A | 0.25 |  |  |
| B | 0.27 |  |  |
| D | 0.22 |  |  |
| Unannotated | 0.26 |  |  |

*Population F_ST_ = 0.31*
