# Supplementary material for: Genetic Dissection of End-Use Quality Traits in Adapted Soft White Winter Wheat
Source: Front Plant Sci. 2018 Mar 9;9:271. doi: 10.3389/fpls.2018.00271 (PMC5861628; doi:10.3389/fpls.2018.00271)
Supplement: Supplementary file 3 [file Table3.docx]

Supplementary Table 3. Number of PCs and covariate used in the mixed model analysis.

| Trait | No. of PCs | Additional Covariate |
| --- | --- | --- |
| Kernel hardness (SKHRD) | 4 | - |
| Kernel size (SKSIZE) | 5 | Glu-D1 |
| Kernel weight (SKWT) | 5 | - |
| Test weight (TWT) | 5 | - |
| Grain protein (WPROT) | 0 | - |
| Break flour yield (BKFYELD) | 3 | - |
| Total flour yield (FYELD) | 3 | - |
| Milling score (MSCOR) | 1 | - |
| Flour ash (FASH) | 3 | Glu-D1 |
| Flour protein (FPROT) | 1 | - |
| Flour SDS^a^ sedimentation (FSDS) | 2 | Glu-D1 |
| Carbonate SRC^b^ (FSRC) | 3 | - |
| Lactic acid SRC (FSRL) | 4 | Glu-D1 |
| Sucrose SRC (FSRS) | 2 | - |
| Water SRC (FSRW) | 3 | - |
| Flour swelling volume (FSV) | 1 | - |
| Mixograph height (MPHT) | 1 | Glu-D1 |
| Mixograph width (MPW) | 2 | Glu-D1 |
| Mixograph width 2 mins (MPW2) | 2 | Glu-D1 |
| Mixograph peak time (MPTIME) | 2 | Glu-D1 |
| Cookie diameter (CODI) | 4 | - |

*^a^ SDS: sodium dodecyl sulfate*

*^b^ SRC: solvent retention capacity*
