# Supplementary material for: Genetic Dissection of End-Use Quality Traits in Adapted Soft White Winter Wheat
Source: Front Plant Sci. 2018 Mar 9;9:271. doi: 10.3389/fpls.2018.00271 (PMC5861628; doi:10.3389/fpls.2018.00271)
Supplement: Supplementary file 7 [file Image1.pdf]

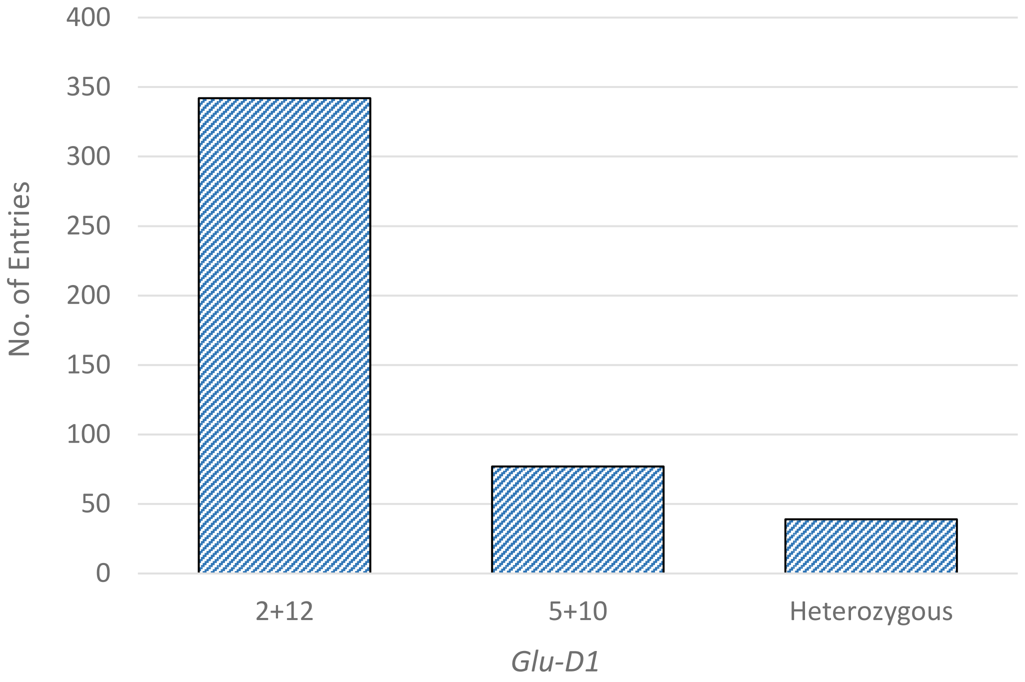


Supplementary Figure 1. Proportion of the elite genotypes carrying a specific allele at each *Glu-D1* loci in the Pacific Northwest soft white winter wheat diversity panel.
