# Supplementary material for: Genetic Dissection of End-Use Quality Traits in Adapted Soft White Winter Wheat
Source: Front Plant Sci. 2018 Mar 9;9:271. doi: 10.3389/fpls.2018.00271 (PMC5861628; doi:10.3389/fpls.2018.00271)
Supplement: Supplementary file 8 [file Image2.pdf]

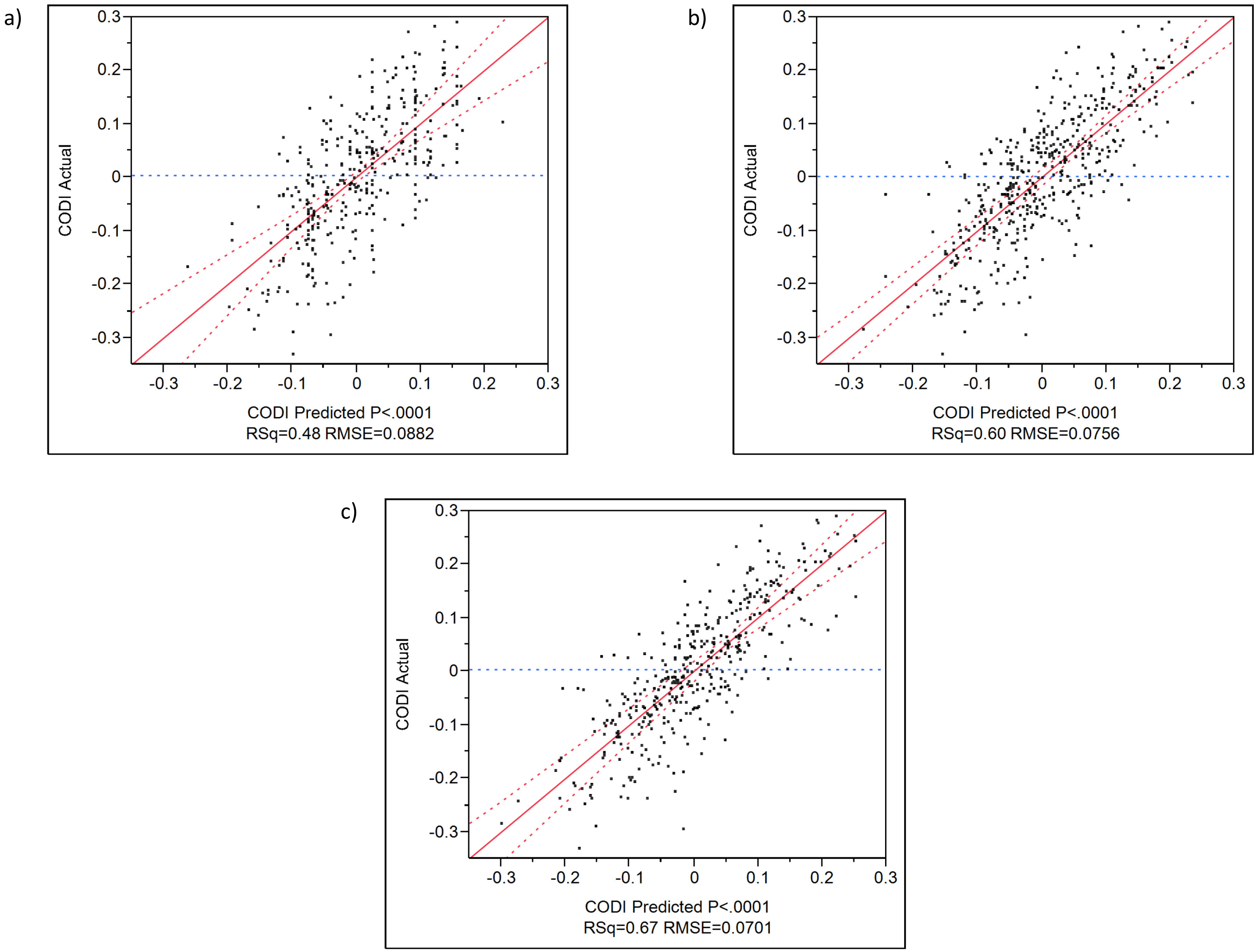

Supplementary Figure 2. Modelling cookie diameter using only QTLs (a), phenotype data (b) and QTL + phenotype data (c) in panel of soft winter wheat. The quality traits and their respective QTLs used include: single kernel hardness, cookie diameter, break flour yield, total flour yield, flour ash content, lactic acid SRC, water absorption SRC and carbonate SRC.
